# Malaysian adolescent students' needs for enhancing thinking skills, counteracting risk factors and demonstrating academic resilience

**DOI:** 10.1080/02673843.2014.973890

**Published:** 2014-10-29

**Authors:** Seffetullah Kuldas, Shahabuddin Hashim, Hairul Nizam Ismail

**Affiliations:** ^a^ School of Educational Studies, Universiti Sains Malaysia, 11800 Penang, Malaysia

**Keywords:** Malaysia, adolescent student, thinking skills, academic resilience, resilience asset, risk factor

## Abstract

The adolescence period of life comes along with changes and challenges in terms of physical and cognitive development. In this hectic period, many adolescents may suffer more from various risk factors such as low socioeconomic status, substance abuse, sexual abuse and teenage pregnancy. Findings indicate that such disadvantaged backgrounds of Malaysian adolescent students lead to failure or underachievement in their academic performance. This narrative review scrutinises how some of these students are able to demonstrate academic resilience, which is satisfactory performance in cognitive or academic tasks in spite of their disadvantaged backgrounds. The review stresses the need for developing a caregiving relationship model for at-risk adolescent students in Malaysia. Such a model would allow educators to meet the students' needs for enhancing thinking skills, counteracting risk factors and demonstrating academic resilience.

## Introduction

During the adolescence period of life between puberty and maturity, students can be subjected to suffering from various challenges, such as neglectful or conflictual relationships with parents, teachers, friends or peers. Although this hectic period is not necessarily universal and inevitable (Eccles et al., 1993), many Malaysian adolescent students suffer from such challenges in one way or another. Hashim (2007) contended that

Malaysian teenagers, in general, face various life challenges and experience distress. They are at risk of becoming dissatisfied and unhappy teenagers and may choose to become involved in negative activities such as gangsterism, bullying, drug abuse, sexual misconduct, and crime (p. 112).

The majority of adolescent students who are exposed to such risk factors in Malaysia (Tan et al., 2012) or in many other countries (Doll, Jones, Osborn, Dooley, & Turner, 2011; Flouri, Tzavidis, & Kallis, 2010; Hanewald, 2011) demonstrate failure or suboptimal performance in cognitive or academic tasks. Due to the failure in acquiring and applying cognitive skills, students even without disadvantaged backgrounds may also be at risk of being unable to perform optimally in future cognitive tasks. Empirical evidence indicated that students' thinking skills, especially the critical thinking, in Malaysian public institutions of secondary (Nagappan, 2000) and higher learning (Nagappan, 2010) were below the expected proficiency level. A perusal review of literature on teaching thinking skills (Kuldas, Allahyar, Hashim, Ismail, & Samsudin, 2014) argued that the suboptimal task performance of the students should not be ascribed solely to the incompetence of educators in teaching how to think. The disadvantaged backgrounds of students should also be taken into account to make a comprehensive evaluation of their task performance (Hanewald, 2011).

Disadvantaged backgrounds that increase the likelihood of an unfavourable experience or outcome are referred to as risk factor (Durlak, 1998). Risk factors are related to: (a) the individuals themselves – underdeveloped communication skills, substance abuse, sexual abuse and risks related to underage sexual intercourse; (b) their family background – poor parental supervision, unemployment, divorce, domestic violence and low socioeconomic status; (c) their exposure in school – feeling of detachment, academic failure and negative influence of peers; and (d) the environment in their community – cultural, racial or gender discrimination, and lack of concern and social support. In particular, a neglectful relationship is a common risk factor. Relying on their longitudinal studies, Cicchetti and Manly (2001) and Lansford et al. (2002) concluded that children suffering from neglectful relationships are at risk of depression, delinquency and academic failure during the period of childhood, adolescence and adulthood.

Despite risk factors preventing the majority of students from succeeding, some with the same background can be academically successful (Morales & Trotman, 2004). This exceptional academic achievement is defined as academic resilience, unlike psychosocial resiliency, which is determined by how emotionally stable individuals might be or how well they adjust themselves to a given environment while facing adversity (Morales, 2008). Benard (1995) argued that academic resilience requires the promotion of its internal assets, which are referred to as problem-solving skills, social competence, critical consciousness, a sense of purpose and autonomy. Problem-solving skills encompass critical, creative and reflective thinking that are practised to identify, formulate and solve problems correctly. Social competence relies heavily on the ability (i.e. communication skills and empathy) to approach people from different cultures, to understand feelings and problems of others, and to elicit positive responses from them. Critical consciousness refers to reflective awareness of the source and structure of risk factors. A sense of purpose is associated with achievement motivation, optimism, persistence and educational aspirations. Finally, autonomy is a strong sense of his or her own identity as well as the ability to exert some control over his or her environment and cognitive tasks.

An effective way to foster the internal resilience is to provide at-risk students with a caregiving relationship (Masten, Best, & Garmezy, 1990). Abdul Kadir et al. (2012b) argued that Malaysian adolescent students who are at risk (e.g. educational failure, delinquency), require a caregiving relationship with an individual who helps them with love, care and attention. A caregiving relationship means that an at-risk adolescent will always be under the attention of someone who genuinely cares for who he or she is (e.g. listening to him or her). At-risk adolescents need a safe relationship or environment to trust and to be trusted, to love and to be loved, to respect and to be respected, and to meet human basic needs (food, drink and shelter) so that they can develop and demonstrate resilience (Hanson & Kim, 2007).

Caregiving relationships with significant individuals in family (siblings or parents), school (peers or teachers) and community (neighbours or friends) environments are potential external resilience assets, as evidenced by a growing body of literature (e.g. Abdul Kadir et al., 2012a, 2012b; Benard, 2004; Benson, Leffert, Scales, & Blyth, 2012; Doll et al., 2011; Flouri et al., 2010; Garmezy, 1985; Luthar, Cicchetti, & Becker, 2000; Masten & Coatsworth, 1998; Masten et al., 1990; Rutter, 1987; Werner & Smith, 1992). According to these literatures, in contrast to the risk factors, the external protective factors are related to positive aspects of the same variables: (a) the individuals themselves – effective social skills, satisfactory academic achievement and secure attachment to family; (b) their family background – caring parents and parental employment; (c) their exposure in school – a sense of belonging, achievement recognition and opportunities for success; and (d) the environment in their community – access to a social support, social networks. In particular, the caregiving relationship with at-risk adolescents helps counteract or protect against risk factors, promote internal resilience assets and stimulate towards academic success.

Although at-risk adolescent students showing academic resilience are a minority; the question is what enables them to perform the same task better than the majority having the same background. In particular, how are Malaysian at-risk adolescent students enabled to construct, develop and demonstrate academic resilience? This issue should be brought to light in order to develop a resilience-enhancing approach for those at-risk adolescents who are still non-resilient academically (Russo & Boman, 2007). This narrative review aims to provide insights into the effects of risk and protective factors on cognitive and academic performance of adolescent students in Malaysia, and, thus, sets out reasons for developing an academic resilience model for the students. The review consists of three main sections. The first section presents some educational endeavours that are aimed at the promotion of students' thinking skills in Malaysia. The next section draws attention to risk factors that Malaysian adolescent students suffer from and are affecting their performance in cognitive or academic tasks. The last section stresses the need for an academic resilience approach to enhancing cognitive performance of the students. The review further highlights the need for further studies on how the resilience assets affect the students' performance and how to help those who are academically non-resilient. The review herby explains how the resilience approach may contribute to the vision of the Ministry of Education Malaysia.

## Promoting thinking skills of adolescent students in Malaysia

Educational endeavours are mainly aimed at moulding students into resilient learners (critical and creative thinkers, effective problem solvers) so that they can gain admittance to better living opportunities and advanced education. Resilience can be (a) the process of human development (Benard, 1991), (b) the developmental capacity with respect to individual differences in responding to risk factors (Rutter, 1990) and (c) the outcome as a quick recovery from an experienced risk factor (Hanewald, 2011). A desired change in the process, capacity and outcome of human development requires teaching students how to acquire and apply thinking skills. Meeting their motivational needs, such as achievement-goal motivation, is also necessary (Kuldas et al., 2014). Thinking skills alongside motivational factors are the core internal resilience assets that need to be boosted. Thus, a variety of challenges in cognitive, emotional, societal, personal or educational aspects human development can be ameliorated.

The promotion of thinking skills is central to the educational philosophy and policy of many countries. The integrated curriculum for Malaysian secondary schools requires every teacher ‘to use teaching-learning methods and techniques which will stimulate, encourage, and develop the thinking abilities of students’ (Curriculum Development Center, 1989, p. 27). The Ministry of Education Malaysia (2013) has recognised the aim of equipping Malaysian students with thinking skills (i.e. rendering them intellectually rigorous, emotionally stable and academically resilient) as central to all their endeavours in order to actualise the vision of the national education philosophy.

To prepare public school teachers in Malaysia, a number of workshops, programmes and short courses on critical thinking have been conducted since the 1980s; but training them in the teaching of thinking skills more explicitly started at teacher education colleges in 1994 (Nagappan, 2001). The thinking skills programme is basically adopted from a model developed by Robert Swartz and Sandra Parks at the National Centre for Teaching Thinking in Boston; therefore, it is called as the ‘Boston Model’ in Malaysia (Educational Planning and Research Division, 1994). Instead of applying a pre-packaged curriculum or programme, the Boston Model suggests the ‘infusion approach’ – integrating thinking skills into all teaching subjects – following four components, namely introduction to content and process, thinking about thinking, active thinking and thinking application (Swartz & Parks, 1994). According to Swartz and Parks, these components allow the teaching of the same skills in distinct subjects at all grade levels. In order to suit the local needs, the Teacher Education Division made an additional component (i.e. consolidation and enrichment activities). All these efforts raise the question: has the objective been achieved optimally or satisfactorily?

### Achievement in teaching and learning of thinking skills

Programmes and approaches for the teaching of thinking skills meet with several criticisms. On one hand, the intervention programmes are inaccessible to the majority of students (Warburton & Torff, 2005). On the other hand, by no means, they assure students of the transference of thinking skills to a new context (Kuldas et al., 2014). For example, a subject-specific approach to teaching problem-solving skills provides little help for how to deal with a problem but rather facilitates problem identification (Ruggiero, 1995). Hu et al. (2011) designed a curriculum for teaching primary school students how to think in a specific context and how to transfer or apply the knowledge to a new context. The design was based on the strengths of the programmes and approaches as well as aimed at stimulating interest in a domain-specific subject. Nevertheless, the curriculum failed after a 4-year intervention. Even most of the participants were still unable to compare and classify targeted concrete concepts. Furthermore, there was no significant effect on low achievers' performance. As Lipman (1985) argued, the subject-specific approach remains promising the skills transference.

According to Hu et al. (2011), the abovementioned failure is mainly due to teachers' lack of knowledge about thinking skills, particularly in teaching novice students and low achievers. A considerable number of studies suggested similar conclusion. For instance, Stapleton (2011) and Zohar (2004) reported that most teachers, who were trained to enable students think critically, had insufficient understanding of what critical thinking means. This lack of understanding was ascribed to suboptimal performance in critical thinking skills of students from various countries, such as China (Tian & Low, 2011), Singapore (National University of Singapore, 2003), Israel (Zohar, 2008), Saudi Arabia (Al-Qahtani, 1995), and the United States (Marin & Halpern, 2011). Highlighting the required understanding, Marin and Halpern (2011) affirmed that most of the trained teachers inadequately prepare adolescent students for demands of cognitive tasks inside and outside the classroom environment.

Although the Ministry of Education Malaysia (2013) centralised the role of teachers in moulding students into critical and creative thinkers, a considerable percent of them are inadequately trained to teach thinking skills. Nagappan (2001) described that 41% of secondary school teachers from Malaysian public educational institutions did not receive any of the training, nor did training the rest 59% bring a significant improvement on their perceptions of teaching thinking skills (i.e. beliefs in their own pedagogical knowledge, skills and attitudes). Mahyuddin, Pihie, Elias and Konting (2004) similarly claimed that many secondary school teachers in Malaysia are not effective enough to incorporate thinking skills in their teaching strategies. Attributable to the teachers' inadequate preparation, the secondary school students' critical thinking skills occurred below the expected proficiency level (Nagappan, 2000, 2001). Nagappan (2000) stated that ‘after 12 or 13 years of public education, many students are unable to give evidence of a more than superficial understanding of concepts and relationships that are fundamental to … subjects they have studied’ (p. 1). Relying on more recent findings, Nagappan (2010) emphasised the need for a comprehensive review of educational programmes for the teaching of thinking skills in Malaysian educational institutions.

However, solely considering educators responsible for students' underachievement leads to a questionable evaluation. Casting light on the role of teachers overshadows the role of students – what role do Malaysian students play in their suboptimal achievement? A comprehensive or convincing evaluation of students' cognitive performance requires shedding light on negative emotional states (e.g. fear of failure), lack of motivation (e.g. low interest) and risk factors (e.g. low socioeconomic status) they experience. Other factors such as students' perceptions and teacher–student interaction patterns, on the acquisition and application of the recommended skills need to be taken into account, too (Kuldas et al., 2014).

As reported on undergraduate students' perspectives on the recommended cognitive skills (Devadason, Subramaniam, & Daniel, 2010; Nikitina & Furuoka, 2012), the students believe that educators alone cannot enable them to acquire and apply knowledge and skills, as their own endeavours crucial as well in this process. However, the scarcity of evidence for such perceptions is attributable to the inconclusive evaluation of the underachievement. Nikitina and Furuoka (2012) found that the literature on the recommended skills leave the students' perspectives largely unclear. What cognitive skills do Malaysian students perceive to be necessary for the acquisition and application? Due to the scarcity of evidence, educators provide the students with less helpful guidance on recognising what cognitive skills (assess their strengths and weaknesses) and how they can acquire and develop (Nikitina & Furuoka, 2012). As an evaluative review (Shakir, 2009) suggested, further studies are needed to identify Malaysian students with lacking in cognitive skills proficiency in order to organise special courses that would help them realise their strengths and weaknesses, and ultimately enhance their cognitive performance.

In addition, the programmes and approaches for teaching of thinking skills largely focus on the cognitive domain (e.g. metacognitive skills, self-awareness and volitional attention), thus, leave little room for the affective domain that includes desires and fears of students (Kuldas et al., 2014). Affective factors, especially negative (e.g. sadness and hopelessness) and positive emotions (e.g. task enjoyment or hopefulness), mould teacher–student interaction patterns, thereby steering the process of teaching and learning thinking skills (Kuldas et al., 2014). The efforts should also be aimed at stimulating student interest in the acquisition and application of thinking skills (Hu et al., 2011). Negligence of the affective domain would lead to provide educators (from Malaysian secondary and higher education) with insufficient insight into students' perspectives and teacher–student interaction patterns, thereby providing inadequate help in establishing a caregiving relationship with their students.

As a result, a student may be exposed to multiple risk factors while growing up, and therefore, be disengaged in learning and teaching activities or be affected emotionally (hopelessness or depression) and physically (deteriorating appearance or self-harming). An at-risk student suffers from aggregated effects of multiple risk factors more than a specific risk factor (Hanewald, 2011; Masten et al., 1990). A single risk factor usually brings about the modest inhibitory effect on students' performance, academic underachievement (Appleyard, Egeland, Van Dulmen, & Srouge, 2005; Fergusson, Horwood, & Lynskey, 1994; Oades-Sese, Esquivel, Kaliski, & Maniatis, 2011). In comparison to those having multiple protective factors, students suffering from various risk factors have different needs to meet academic success. Not every educator or institution can meet all the varying needs of at-risk students. Moreover, most educators may be unaware of the inhibitory effect of risk factors or recognise resilience levels of their students; yet, those having the awareness may have little or no opportunity to control or ameliorate the effects or to deal with risk factors (Russo & Boman, 2007). An educator or educational institution may provide developmental supports that promote academic success, but may not be able to eliminate every risk factor or the bulk of risk factors that promote failure. Due to the failure in enabling adolescent students to acquire and apply thinking skills, they can be at risk of being unable to meet challenges in cognitive tasks inside and outside the school environment.

## Are Malaysian adolescent students at risk?

In 2010, the Malaysian adolescent population (10–19 years old) was estimated to be 5.5 million (UNICEF, 2010), while the Malaysian youth demography (15–25 years old) was numbered around 5.2 million (Department of Statistics Malaysia, 2010), which is approximately 19% of the total population. Relying on the World Youth Report, Abdul Kadir et al. (2012b) estimated that 25% of the population would be classified as at-risk youth. According to the Malaysian Youth Report (Hamzah, 2007), substance abuse and underage sexual intercourse are the most prevalent risk behaviour among the adolescents and youths.

A series of recent studies drew attention to the increased crime rate, such as pornography, destructive behaviour, truancy (Mey, 2009, 2010), and drug addicts (Ghani, Zamani, Rahman, Zainal, & Sulaiman, 2008; Mohamed, Marican, Elias, & Don, 2008) among Malaysian youths, thereby raising the concern over juvenile delinquency (Nasir, Zamani, Khairudin, & Wan Shahrazad, 2011; Nasir, Zamani, Yusooff, & Khairudin, 2010). Family, peer and school environments directly or indirectly contribute to these risk behaviours (Nasir et al., 2011). In particular, negligence, as an absence of a caregiving relationship with adolescents, is a significant risk factor underlying the development of risk behaviours. Related studies showed that most of the drug addicts started abusing substance when they were still secondary school students (Ghani et al., 2008). Malaysian teenagers perceived negative parental attitude as the factor leading to drug abuse, associating their actions with their unfulfilled needs for respect, love and fair treatment from their neglecting parents (Low, Zulkifli, Yusof, Batumalail, & Aye, 1996). In a further research (Low, Ng, Fadzil, & Ang, 2007), Malaysian adolescent boys (13–17 years old) ascribed their involvement in sexual intercourse to tension and pressure from family. On the other hand, another study (Zulkifli & Low, 2000) showed that adolescents who were free from parental control (i.e. living away from their parents) appeared to have more experience of sexual intercourse, which could be due to the pressure of peers or social groups. Thus, risk factors in all these cases are attributable to the absence of a caregiving relationship with parents, friends or peer groups (i.e. the lack of external protective factors).

Talib, Mamat, Ibrahim and Mohamad (2012) asserted that there are a considerable number of sexually active teenagers, teenage pregnancy and illegitimate children in Malaysia. Concern over these social issues is growing in the country (see Low, 2009; Shahabudin & Low, 2013; Tan et al., 2012). According to reviewed studies by Talib et al. (2012), 43% of youth respondents (all from the Malay community) started to have dates as early as their ages of 13–15. At the age of 16–17, 35% of them begun to kiss and caress their partners. As reported by the Health Ministry of Malaysia (Talib et al., 2012), 54% of youth participants (17–24 years old) had more than one sexual partner. Relying on further evidence obtained from the National Registration Department, Talib et al. proclaimed that around 234,647 illegitimate infants were born out of 2 million births between 2006 and 2009 years in Malaysia. Yet, the prevalence of teenage pregnancy is increasing in the country (Tan et al., 2012). The prevalence among 4500 teenagers (12–19 years old) increased to 5.4% in the state of Negeri Sembilan (Lee, Chen, Lee, & Kaur, 2006). A similar study (Anwar, Sulaiman, Ahmadi, & Khan, 2010) reported a much higher prevalence; 12.6% among 1139 students (15–20 years old), in the state of Penang alone. These risk factors are mostly attributable to neglect (e.g. family is not a source of strength or not close enough), unemployment, and lower levels of educational and socioeconomic status (Omar et al., 2010; Tan et al., 2012).

In a recent study (Nik Farid, Che'Rus, Dahlui, & Al-Sadat, 2013), the strongest predictor of underage sexual intercourse appeared to be a history of sexual abuse during the childhood of incarcerated adolescents (aged 12–19 years) in Malaysia. This was followed by past experience in alcohol and illicit drug abuse, as well as pornography. Participants of the study consisted of 1049 incarcerated adolescents from more than half of all welfare institutions within the 11 states in peninsular Malaysia. Among these adolescents, 654 with the mean age of 14 (range 8–19) reported sexual intercourse. Nik Farid et al. (2013) stressed that child abuse generates deeper sense of worthlessness, and thus, associates with lower self-esteem and higher rates of depression. In a study by Abdul Kadir and Desa (2013), Malaysian female university students who suffered from depression reported an experience of physical and sexual abuse as well as parental antipathy and neglect during childhood.

Physical (e.g. slapping face, head or ears) and emotional (e.g. threatening, insulting, or embarrassing) violence (Kasim, Shafie, & Cheah, 1994), especially sexual violence against children may be at a higher level than reported in Malaysia (Choo, Dunne, Marret, Fleming, & Wong, 2011). A total of 6.8% of 616 respondents, consisting of Malaysian student nurses and medical assistant trainees, informed about their childhood experiences of sexual abuse (Singh, Yiing, & Nurani, 1996). A meta-analysis of prevalence rates of child sexual abuse across countries indicated that 8.3% of Malaysian female respondents had suffered some form of sexual abuse before their age of 18 (Pereda, Guilera, Forns, & Gómez-Benito, 2009). This prevalence rate is, however, likely to be higher in the present time as the meta-analysis has relied on only one early study by Singh et al. (1996).

Kassim and Kasim (1995) found that child sexual abuse is significantly associated with family-related risk factors (i.e. neglect, unemployment and lower levels of educational and socioeconomic status). However, the loss of some traditional values, according to which parents used to bring up their children, could also be a reason for an increase in child sexual abuse (see Lalor, 2004). Raybeck and De Munck (2010) argued that Malaysian traditional values, such as showing concern for others and the social networks within the community, are attenuated by modernisation. Raybeck highlighted that, for the first time after conducting many studies on the traditional values of Malay communities, he encountered villagers who did not know the names of their neighbours.

Traditional values could allow neighbours act on the part of parents in observing and correcting a maltreatment or abuse. According to Abdul Kadir et al. (2012b), a caring neighbourhood climate can be a significant predictor of socially desirable behaviour. Such a neighbourhood, moreover, may consolidate the feeling of belonging to the community. In traditional cultures advocating the neighbourhood climate, violence against others or even against oneself tend to be lower but may occur more and more as globalisation encourages the sentiments of individualism within one's values (Arnett, 1999). The loss of traditional values might be conceived as the loss of caregiving relationships. Choo et al. (2011) found that one in every three Malaysian adolescents (15–17 years old) had multiple experiences with various types of violence, which they strongly associated with the lack of caregiving relationship with parents as well as individuals in their schools and neighbourhoods.

Thus, family-, community- and individual-related risk factors are attributable to academic underachievement and dropping out of school in Malaysia (Tan et al., 2012). To protect children and adolescents against such risk factors, Nik Farid et al. (2013) suggested developing innovative programmes such as workshops on parenting skills to help caregivers grasp the significance of nurturing their children. For a similar suggestion, Weatherley et al. (2012) highlighted the paucity of comprehensive studies on the sexual abuse of children in Malaysia and drew attention to the need for a school-based sexual abuse prevention curriculum (see also Choo et al., 2011). For better innovative programmes or effective curriculum, further investigations on the sexual abuse of children as well as on the sexual tendencies of adolescents are needed. The risk and protective factors relevant to academic resilience could be considered.

According to Hashim (2007), ‘the increasing social problems among teenagers in Malaysia are, in fact, a manifestation of their inability to cope with the challenges of everyday life’. Therefore, ‘it is essential to understand the type of problems teenagers in Malaysia are facing and whether or not they are equipped with adequate coping skills to deal with these challenges’ (p. 98). Hashim displayed that 34.9% out of 209 respondents (Malaysian adolescent students, 16 years old in average) experienced various forms of distress at home (e.g. disputing with siblings and parents); 31.5% at school (e.g. being in conflict with peers and teachers, being hit and embarrassed by them); and 77.0% in relation to difficulties in academic subjects (e.g. Mathematics, Physics, English Language and History). A similar study, by Wahab et al. (2013), focusing on total 360 secondary boarding school students (16 years old in average) in Malaysia, revealed that the prevalence of stress, anxiety and depression was slightly higher (39.7%, 67.1% and 44.9%, respectively) compared with previous studies (see Ramli et al., 2008; Yusoff, 2010; Yusoff et al., 2011). These findings suggest that low socioeconomic status (see also Ong, Chandran, Lim, Chen, & Poh, 2010), high academic pressure and the lack of parental support are significantly linked to the risk factors that may lead to academic failure or poor performance as well as disciplinary problems at school. The inability to deal with stress alongside with socioeconomic disadvantaged backgrounds and peer pressure appeared to be the major reasons for substance use (Baharudin, Krauss, Yaacob, & Pei, 2011). Similar reasons are also attributable to underage sexual intercourse and teenage pregnancy (Tan et al., 2012).

As a result, to avoid or control the abovementioned risk behaviours, further investigations are needed to develop an optimal resilience-enhancing model for non-resilient Malaysian adolescent students. Prospective findings may help describe and explain the internal and external factors that enable some of the at-risk adolescent students to be academically successful in spite of obstacles that prevent the majority with the same background from demonstrating academic resilience. Future studies should be aimed at identifying Malaysian teenagers who are at-risk, developing a resilience model and subsequently designing programmes that can facilitate and foster the development of internal and external resilience assets.

### Future directions for better teaching of thinking skills: the need for an academic resilience approach

A noteworthy finding in the study by Hashim (2007) indicated that the teenagers who reported high academic achievement had a greater number of stressful conflicts or problems. This finding, however, leaved unclear what enabled the adolescent students to attain the satisfactory achievement in academic tasks. In case of facing conflicts at home, some of the students stated they talked to friends to feel better. As for the conflicts at school, talking with peers, teachers, parents or counsellors was a coping strategy. Regarding to difficulties in academic subjects, the students tended to have peer-group discussion, help from elder siblings or tutors and advice from teachers. The students noted that their parents provide more problem-focused supports in terms of advice and finance, but not an optimal emotional support; their friends are better in providing emotional supports, whereas their teachers are helpful in dealing with issues related to school or academic subjects. Thus, as Hashim (2007) noted, how these supports (protective factors) contribute to academic achievements need to be explained further.

According to ‘Resilience Theory’ (Rutter, 1987; Ungar, 2005) and ‘Educational Resilience Theory’ (Wang, Haertel, & Walberg, 1994), multiple levels of the surrounding environment, such as family, school, societal and neighbourhood, mould the process of human development and human behaviour as individuals grow within a complex system of relationships. ‘Resilience does not come from rare and special qualities, but from the everyday magic of ordinary, normative human resources in the minds, brains and bodies of children, in their families and relationships, and in their communities’ (Masten, 2001, p. 9). Resilience is not a fixed individual quality or an individual trait that one has or has not (Zimmerman & Arunkumar, 1994) but is ‘an inborn developmental wisdom that naturally motivates individuals to meet their human needs for love, belonging, respect, identity, power, mastery, challenge, and meaning’ (WestEd, 2002, p. 2).

Students who are provided with a caregiving relationship may demonstrate satisfactory academic achievements despite their disadvantaged backgrounds (Borman & Overman, 2004; Martin & Marsh, 2006). Stemming from Ecological Systems Theory (Bronfenbrenner, 1979), the Educational Resilience Theory (Wang, Haertel, & Walberg, 1999) takes into account the ecological perspective; it proposes that caregiving relationships between individuals in family, school or social community need to be established so that students can demonstrate academic resilience. According to the resilience theory, students may confront adversities anywhere or at any time, whereby they would need to draw on the resilience assets developed in their environments and within themselves to counter risk factors. As a developmental process, resilience assets empower individuals to mould an environment they are in, which in turn mould their personal and cognitive development. This is a widely accepted ecological framework for understanding the dynamic interactions between risk and protective factors (see Doll et al., 2011; Esquivel, Doll, & Oades-Sese, 2011; Gordon & Song, 1994; Morales & Trotman, 2004; Von Soest, Mossige, Stefansen, & Hjemdal, 2010).

Adolescents essentially draw on their internal resilience assets, but the external resilience assets allow them to develop and demonstrate resilience, thus, facilitate altering or even reversing expected negative outcomes (Garmezy, 1985; Jain, Buka, Subramanian, & Molnar, 2012; Mandleco & Craig, 2000). The external assets enable them to become self-reliant and empathic, thereby approaching people and circumstances trustfully and hopefully (Grotberg, 1995). Under adverse situations, a cumulative impact of the external resilience assets increases positive outcomes. For instance, it facilitates the process of coping with adjustment problems (Rutter, 1999) or the avoidance of failure (Jain & Cohen, 2013; Oades-Sese et al., 2011; Rutter, 1984; Werner, 1993; Werner & Smith, 1992).

To enhance the internal resilience, family usually provides the most caregiving environment, whereas peer and school environments are associated with an important increase in the resilience level (Brooks, 2006). Via a caregiving relationship with a student having low academic resilience, teachers may promote the internal resilience assets (Werner & Smith, 1992). Teachers' relationships with at-risk adolescent students need to be based on reciprocity and collaboration (i.e. sharing power). Instead of controlling and competing, the students can be given a role to participate in teaching and learning activities. Their intrinsic motivation and learning ability can hereby be improved (Benard, 2004). Withholding such opportunities would lead to detachment of the at-risk students from teachers and curricular activities (WestEd, 2002).

As a result, the academic resilience approach to students' performance in cognitive and academic tasks is based on the basic tenet that everyone is born with resilience as the process and capacity of human development. Strengthened resilience enables adolescents to rebound from adversity as well as to succeed in spite of it. The resilience approach proposes focusing on how some adolescents thrive against all odds (i.e. examining protective factors that promote one's resilience, academic success, instead of merely focusing on how to eliminate risk factors). However, although it is not necessary to promote all the resilience assets, to focus merely on developing, one of them is not very effective in rendering students sufficiently resilient (Grotberg, 1995). For instance, adolescent students may have a loving and warm relationship with parents or teachers, but lack the necessary self-awareness of their own thoughts and feelings. Self-awareness is also not enough to build sufficient resilience when they have nobody to help them or do not know how to communicate with others in order to solve problems, which they could not handle on their own. Effective resilience requires a combination of both internal and external assets (Willms, 2002). A further study could draw on the academic resilience approach to identify internal and external resilience assets that need to be promoted, thereby explaining how Malaysian adolescent students develop and demonstrate their academic resilience.

## Conclusion

To have resilient and intellectually rigorous students (critical and creative thinkers, efficient problem solvers) is a central objective to educational endeavours in Malaysian secondary and higher education. This narrative review has collated findings that are associated with underachievement of this objective, as students from Malaysian public institutions of secondary and higher learning are found to be lacking the expected proficiency level of performance in cognitive tasks. The review has argued that educators cannot solely be responsible for this unsatisfactory result, as the various risk factors that the students suffer from should also be taken into account. The reviewed literature has confirmed that risk factors that are associated with the individual themselves (e.g. drug abuse, sexual abuse and sexual misconduct), their family background (e.g. neglect and low socioeconomic status), their exposure in school (e.g. a sense detachment, academic failure and teachers' insufficient understanding) and the environment in their community (e.g. lack of social support and discrimination) usually lead to failure or poor performance in cognitive or academic tasks. However, in spite of obstacles that prevent the majority of students from succeeding, some students with the same background can demonstrate academic resilience. How Malaysian adolescent students are able to have academic resilience is unclear. Empirical data concerning how the students construct academic resilience have yet to be provided.

Prospective studies on the Malaysian context could have several objectives: First, to identify the internal and external resilience assets of Malaysian adolescent students; second, to predict the best external resilience assets that promote internal resilience; third, to determine the best internal assets that are associated with satisfactory performance in cognitive or academic tasks. The fourth objective is to determine relationships between the resilience assets and an approach (e.g. infusion approach) to teaching thinking skills. To facilitate the actualisation of the educational objective, prospective findings could suggest a model of academic resilience for Malaysian adolescent students, showing the relationship between the resilience assets and the approach to teaching of thinking skills. In particular, how a caregiving relationship meets the students' affective/motivational needs and be conducive to their academic resilience could be explained in the model.
